# Recapitulating the immune microenvironment in pediatric brain cancer: preclinical modeling strategies

**DOI:** 10.3389/fimmu.2025.1642668

**Published:** 2025-10-01

**Authors:** Adip G. Bhargav, Joseph S. Domino, David Akhavan, Jo Ling Goh

**Affiliations:** ^1^ Department of Neurological Surgery, University of Kansas Medical Center, Kansas City, KS, United States; ^2^ Division of Neurosurgery, Department of Surgery, Children’s Mercy Hospital, Kansas City, KS, United States; ^3^ Department of Radiation Oncology, University of Kansas Medical Center, Kansas City, KS, United States

**Keywords:** pediatric brain cancer, preclinical modeling, immunotherapy, glioma, medulloblastoma, tumor microenvironment, organoid

## Abstract

Primary brain and CNS tumors comprise the most common tumors in children and are the most common cause of cancer deaths in this population. Pediatric primary malignant brain tumors including high-grade glioma and medulloblastoma account for a significant proportion of these cancer deaths. Advances in the surgical management and adjuvant treatment paradigms have improved the prognosis of many patients with these tumors, but there remains a subset of treatment-resistant tumors or tumors with unique genetics aberrations and aggressive phenotypes that confer a poor prognosis. Immunotherapeutic strategies have demonstrated promise in pre-clinical studies and early clinical trials. However, high-fidelity evaluation of these novel therapeutics and subsequent clinical translation has faced challenges due to the limitations of conventional preclinical models that have been used to study the pathophysiology and treatment response of these tumors. Recent efforts have been directed towards more accurate modeling of the molecular and histological heterogeneity observed in these tumors as well as creating immunocompetent animal models that resemble the tumor immune milieu. In this review, we provide an overview of contemporary and emerging preclinical modeling strategies with a focus on those that strive to recapitulate the immune and microenvironment features of malignant pediatric brain tumors.

## 1 Introduction

Pediatric primary brain and central nervous system (CNS) tumors are among the most common tumors to afflict children, and their incidence is comparable to pediatric hematologic malignancies (1, 2). These tumors also account for the most cancer-related deaths in the pediatric population (1). Malignant brain tumors arising from the brain represent a significant proportion of cancer-related deaths and include high-grade glioma, medulloblastoma, choroid plexus carcinoma, among others (1, 2). Current treatment for these tumors primarily involves maximal, safe surgical resection with the possibility of cure with complete removal. In situations where this is not possible due to tumor or patient factors such as location of the tumor or involvement of critical structures, surgical resection is followed by adjuvant radiation therapy and/or chemotherapy (2–4). For certain malignant brain tumors such as diffuse pontine glioma (DIPG), neither surgical resection nor adjuvant therapies are viable options, and novel therapies are needed (2–4). Similarly, in the setting of recurrent or highly aggressive tumor subtypes, available treatments are minimally effective, and prognosis remains poor (1, 2, 4).

**Figure 1:**
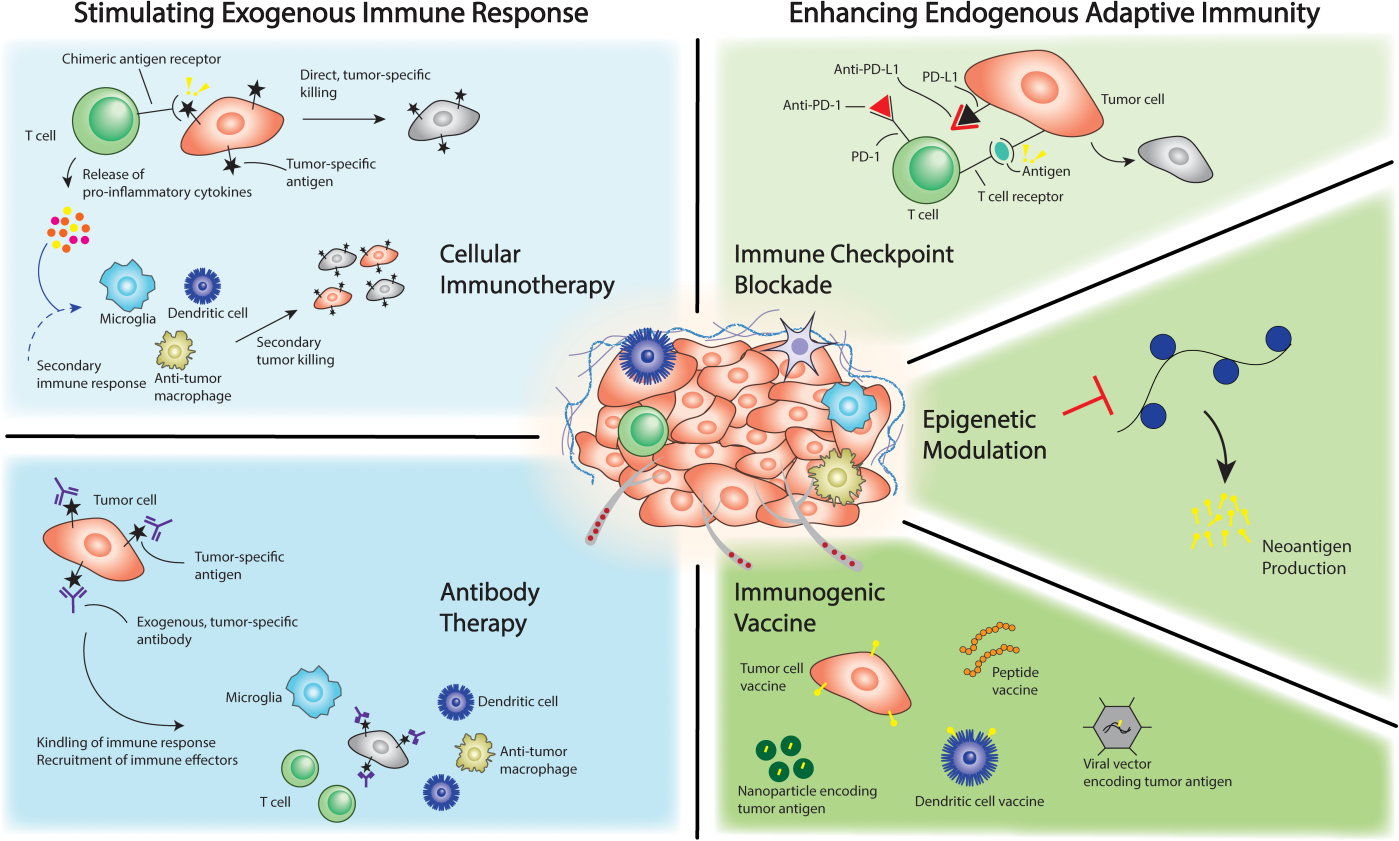
Immunotherapeutic approaches against brain cancer.

The advent of immunotherapy approaches in cancer and the success observed in the treatment of several blood and solid cancers in adults inspired an alternative therapeutic strategy in the treatment of pediatric brain tumors (3, 4). Immunotherapy strategies against brain tumors have continued to evolve over recent years as the understanding of the basic biology and tumorigenesis has improved and represents an area of intense ongoing research (4–6). Current strategies are largely centered around stimulating a patient’s immune system against tumor or the delivery of immune-based, cell therapy that is able to target the tumor (**Figure 1**) (3, 4, 7–9). Specifically, the former strategy aims to galvanize the adaptive immune system against tumor utilizing a number of approaches. These include immune checkpoint point inhibition and enrichment of neoantigens in the tumor milieu through epigenetic therapeutics or tumor vaccines (4, 7, 10). Alternatively, immune-based cell therapy most commonly involves engineering of T cells to express chimeric antigen receptor (CAR) targeted to tumor-associated antigens or other receptors enabling tumor-targeting. In this approach, groups have described local and peripheral delivery of the cell therapy to directly target tumor cells and simultaneously stimulate the adaptive immune system (3, 4, 7, 10, 11). The strategies summarized in **Figure 1** are germane for preclinical modeling of the immune microenvironment because the ideal model would recapitulate key features that are necessary to evaluate and refine these strategies in a preclinical setting. For example, to adequately evaluate the efficacy of cellular immunotherapy approaches, the ideal preclinical model would necessarily utilize a strategy that accurately reconstitutes the immune cellular milieu of the tumor type in question (microglia, dendritic cells, macrophages). Similarly, T cells in addition to the aforementioned cell types would be incorporated into the ideal model when evaluating immune checkpoint blockade and antibody-based therapy approaches. Understanding the mechanism of current treatment strategies in this way can be important in selecting or developing the appropriate preclinical model for therapy evaluation as will be discussed in subsequent sections.

Immunotherapy strategies have displayed promising results in preclinical studies and are undergoing clinical evaluation in several clinical trials of which representative trials are listed in **Table 1** (3, 4, 11–16). The growth of immunotherapy-based clinical trials underlines the need for high-fidelity, advanced preclinical modeling that can accurately recapitulate immune system features and by extension evaluate the efficacy of these therapeutics towards novel immunotherapy development and validation. However, initial results from clinical trials indicate challenges in achieving durable efficacy with these therapies (11, 12, 15–17). In clinical trials studying glioblastoma in adults, immune checkpoint inhibition has not demonstrated efficacy, and questions remain regarding penetration of the therapeutic agent into the tumor milieu as well as immune escape phenomenon whereby the tumor may adapt and alter its immune profile to evade the immune response (3, 4, 11, 14, 16). Additionally, the interaction between the tumor microenvironment, including immune cells and mechanical components such as the extracellular matrix and blood-brain barrier, and immunotherapy is poorly understood particularly in the context of pediatric brain tumors (10, 18–21). These potential barriers to effective clinical translation of immunotherapy are discussed in detail in subsequent sections and warrant further study. There has been significant innovation and new understanding of the basic biology of pediatric brain tumors, and in this review, we highlight advances in preclinical modeling strategies that aim to deconstruct and recapitulate the pediatric brain tumor microenvironment towards testing and designing robust immunotherapeutic strategies. We focus on medulloblastoma and glioma given the large amount of work being done in these areas as well as highlight salient examples from other tumor models. Given the diversity of pediatric brain tumors, we also aim to highlight different challenges encountered with modeling different tumor types and strategies that may be used to overcome these challenges.

**Table 1:** Representative active and recruiting immunotherapy pediatric brain tumor clinical trials.

Immunotherapeutic strategy	Tumor type	Therapeutic agent	Status	Trial identifier
*Immunogenic Vaccine*	MB, Malignant glioma	Cytomegalovirus-specific peptide	Active	NCT03299309
	HGG	Tumor RNA-loaded lipid particles	Active	NCT05660408
	MB, EP, GB, embryonal tumors, astrocytoma	GD207 oncolytic herpes simplex virus-1	Recruiting	NCT03911388
	HGG, DIPG	WT1 mRNA-loaded dendritic cell	Active	NCT04911621
	GBM	Total tumor mRNA and lysosomal-associated membrane protein-loaded liposome	Recruiting	NCT04573140
	MB, metastatic disease	Cytomegalovirus-specific peptide	Recruiting	NCT05096481
*Cellular Immunotherapy*	CNS embryonal tumors	Multi-tumor antigen specific CTL	Recruiting	NCT06193759
	MB, HGG, DMG	iC9-GD2-CAR T cell	Recruiting	NCT05298995
	HER2-positive CNS tumor	HER-2-specific CAR T cell	Active	NCT03500991
	DMG, DIPG, refractory CNS tumor	B7H3-specific CAR T cell	Recruiting	NCT04185038
	DIPG, DMG, refractory CNS disease	B7-H3, EGFR806, HER2, IL-13 zetakine CAR T cell	Recruiting	NCT05768880
	HGG	IL-8R modified CD70 CAR T cell	Recruiting	NCT06946680
*Immunomodulatory agents*	EP, MB, GB	BTK-inhibitor + IDO-inhibitor	Recruiting	NCT05106296
	DIPG, EP, GB	IDO-inhibitor	Recruiting	NCT04049669

MB, medulloblastoma; HGG, high-grade glioma; EP, ependyoma; GB, glioblastoma; DIPG, diffuse intrinsic pontine glioma; DMG, diffuse midline glioma; CNS, central nervous system.

## 2 Current challenges and considerations in modeling pediatric brain tumors

**Figure 2:**
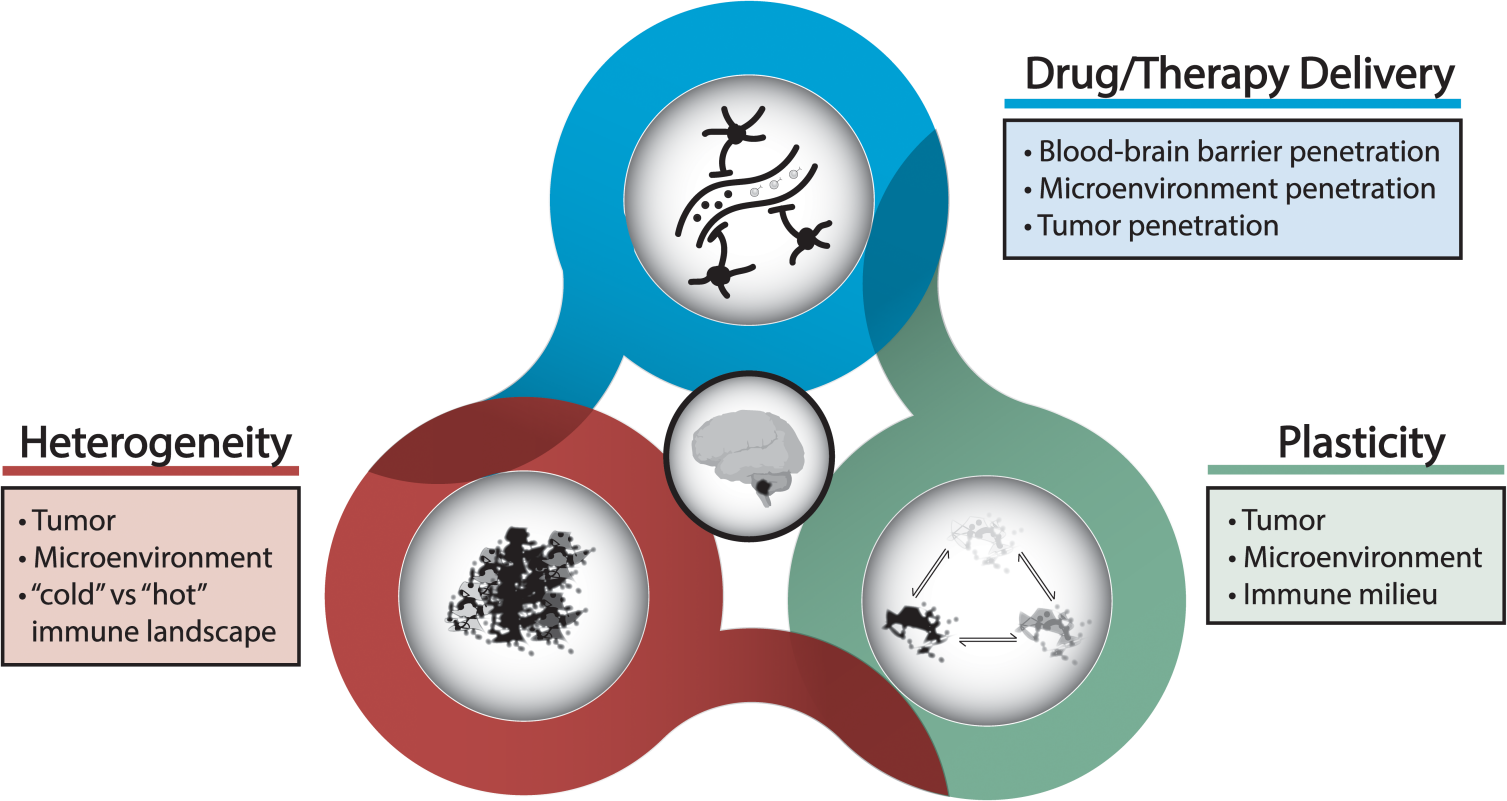
Challenges and considerations underlying treatment resistance and disease progression.

Current understanding of the basic biology of pediatric brain tumors, namely medulloblastoma and glioma, has evolved over the past decades beyond traditional histopathological classifications to include molecular and epigenetic characterization of tumor subtypes (3, 6, 9, 22–28). This work has led to the discovery of the concepts of tumor heterogeneity and tumor plasticity and evolution as it pertains to treatment (**Figure 2**). In the case, of the aforementioned immunotherapy approaches, tumor heterogeneity poses a significant challenge for targeted immunotherapy especially in the case of brain tumors that have been described to be immunologically “cold” or devoid of the immune milieu or cell surface antigens needed to kindle an anti-tumor immune response (21, 24–26, 28–31). As a result, effective immunotherapeutic agents are required to have robust targeting to a tumor-associated antigen or other tumor microenvironment target that is ubiquitous so that it is not affected by heterogenous tumor populations. Similarly, the ideal targeted immunotherapeutic should have the ability to overcome adaptation and plasticity inherent to many tumor cells where tumor cells may downregulate a specific antigen or alter their phenotype to evade immune response, i.e. immune escape or immune evasion strategies (4, 11, 16). In order to develop targeted immunotherapeutic agents or cellular therapies with effective targeting, a model system that accounts for tumor heterogeneity and plasticity as well as incorporates dynamic interactions between other immune cells in the tumor microenvironment is necessary for high-fidelity evaluation of novel therapeutics.

In addition to effective targeting of immunotherapeutic agents to tumor cells, delivery of therapy to the tumor and infiltrating cells poses another unique challenge in the setting of intracranial malignancies. The blood-brain barrier is a feature of pediatric brain tumors that is not seen in systemic tumors which can limit the delivery of immunotherapeutic drugs to the tumor (**Figure 2**). Even in situations where the blood-brain barrier may be disrupted focally by the tumor, it is currently unknown if optimal drug accumulation is seen within all parts of the tumor, and this may underlie cases of inadequate therapeutic response (21, 30, 32). The blood-brain barrier can also pose a challenge to effective crossing of cellular therapies when administered peripherally. Strategies to overcome the blood-brain barrier include transient disruption with adjunctive therapies such as focused ultrasound or chemical agents as well as incorporation of a targeting moiety to the drug construct to facilitate transport (21, 30, 32, 33). Studies have also highlighted diversity in the mechanical properties of brain tumors, particularly gliomas; tumors may exhibit heterogeneous mechanical properties dependent on tumor microenvironment components such as the extracellular matrix as well as the inherent tumor subtype which may ultimately influence penetration of therapeutic agents or cellular therapies (21, 28, 30, 31). Nevertheless, the efficiency of immunotherapy delivery to tumor needs to be tested thoroughly prior to clinical translation, and this can be made possible by employed models that include these components of the tumor microenvironment (18, 20).

**Figure 3:**
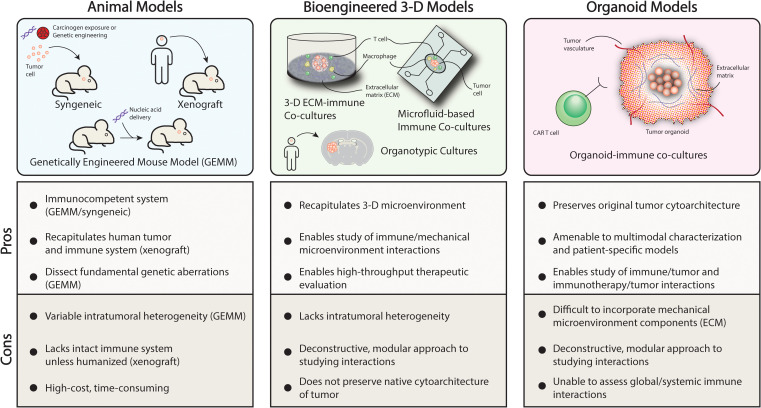
Preclinical modeling strategies to recapitulate the tumor microenvironment. .

Characterization of diverse immune profiles in pediatric brain tumors as well preclinical studies examining the constituents of the tumor microenvironment have revealed heterogeneity and plasticity not only in tumor cells but also surrounding cells in the microenvironment (**Figure 2**). These cellular players include macrophages, neutrophils, and astrocytes, among other cell types that are now understood to play a critical role in immunomodulation within the tumor microenvironment as well as globally (3, 5, 15). One such mechanism is the polarization of macrophages in the tumor milieu towards a pro-tumor phenotype that has been implicated in promoting tumor progression, tumor persistence, and dampen anti-tumor immune responses (3–6, 20, 34). The interaction between these cell types in the tumor microenvironment and immune-based cell therapies has been identified as one obstacle to durable tumor killing due to inactivation mechanisms or T cell exhaustion in the case of CAR-T cell therapies (11, 14, 16, 35). State-of-the-art preclinical models of pediatric brain tumors aim to incorporate dynamic interactions amongst all these cell types to accurately evaluate the efficacy of immunotherapeutic strategies (10, 21, 36–38). In the subsequent sections, advanced preclinical models will be discussed that enable the evaluation of these factors with a focus on animal models and models that mimic the physiological properties of the brain tumor microenvironment (**Figure 3**).

## 3 Engineering animal models to recapitulate the tumor immune microenvironment

Animal models of pediatric brain tumors which are primarily murine models carry several advantages in capturing the complexities of the tumor microenvironment. Compared to cell lines which may be a useful, low-cost means of drug screening or exploratory studies, animal models can be engineered to answer fundamental questions regarding the tumorigenesis and key events surrounding tumor progression or test immunotherapeutic strategies in a system with brain architecture and an immune system that resembles those of human patients (10, 21, 39). At the advent of preclinical animal brain tumor models, the primary goal was to understand features surrounding tumor formation and to try to recapitulate the histopathology of the human disease which will be detailed in some of the representative studies highlighted within this section. However, recapitulation of the tumor microenvironment including the relevant immune features is the focus of recent and ongoing efforts as the importance of the immune system and its interplay with brain tumors has come to the forefront. We highlight recent studies that have been able to replicate features of the tumor immune microenvironment though studies validating the fidelity of these features compared to the human disease are forthcoming (3, 4, 7, 10, 20). Murine models of pediatric brain tumors can be broadly divided into three categories: 1) syngeneic 2) genetically engineered mouse model (GEMM) and 3) xenograft (**Figure 3**). Each type has been employed effectively in order to study different types of therapeutics (10, 32, 40, 41).

Syngeneic models are created by implanting a murine tumor cell line into a mouse line for engraftment. The tumor cell lines are typically created via exposure to a carcinogenic agent or engineering through transposon-based methods and are often widely available and easy to maintain (10, 40, 42–44). The cells may be implanted systemically or orthotopically in the brain of the mouse line to more closely mimic the brain architecture and the blood-brain barrier (10, 40, 41).The primary advantage of this type of model is that a full immune system is preserved which enables *in vivo* testing of immunotherapeutic agents or cell therapies as well as study of the interactions of the therapy with the endogenous immune system (40–42, 45). Importantly, the cell therapies used in these studies are also murine in origin in order to avoid graft versus host reactions. *Scheulke et al.* also demonstrate that syngeneic models of brainstem gliomas with competent immune systems are suitable for assessing neurotoxicity with different types of immunotherapies including CAR T cell therapy which is an important consideration in clinical translation (44). Though this is a system that incorporates most of the key components of the tumor microenvironment as well as the tumor immune microenvironment as previously discussed, ultimately the disease processes being studied are murine in origin and may not accurately reflect human pathophysiology (44). Studies have also revealed that syngeneic models of glioblastoma exhibit variable immunogenicity and may not replicate the immune profile of human tumors depending on the cell line that is used; as a result, immune responses observed in these models must be interpreted with caution and may warrant testing in multiple models (40, 41). However, *Chatinier et al.* report the creation of distinct syngeneic diffuse midline glioma models that accurately recapitulate the immune microenvironment features of patient-derived diffuse midline glioma including intratumoral myeloid cell infiltration and enrichment of tumor-associated antigens (43). They created this model by targeted intra-uterine electroporation in C57BL/6 mice and show that the model is reliable for testing of epigenetic immunotherapeutic agents such as HDAC inhibitors (43). Using Panobinostat as the candidate drug, the group demonstrates that therapeutic sensitivity to treatment is similar to that observed with patient-derived cell lines suggesting that this model is a reasonable proxy for preclinical testing (43). *Khalsa et al.* corroborated similar findings in immunophenotyping various syngeneic glioblastoma cell lines using a combination of RNA-sequencing, cytometry by time of flight, and immunohistochemistry; they demonstrate the immune profiles varied among models but simulated behavior and phenotype of patient-derived samples (42).

GEMMs comprise a distinct group of murine models that are particularly useful for studying the early steps in tumorigenesis as well as drug screening in pediatric brain tumors (10, 41). GEMMs can be created by identifying suspected key driver mutations that are essential for the oncogenesis of a particular type of tumor, and then engineering this genetic driver into mice (10, 46). There are a number of different methodologies used to engineer key mutations or knockouts into murine lines that include crossbreeding, viral vector-based engineering, *in utero* electroporation, or transposon-mediated delivery which are summarized in **Table 2** (10, 47, 48). The traditional and most common method of engineering GEMMs has been through the use of the Cre-LoxP system which can be used to create conditional knockout mice; this system can also be designed to incorporate inducible hormone response elements that enable temporal control over the genetic events of interest (10, 46, 49). The RCAS/t-va is a prominent alternative to the traditional Cre-LoxP system that employs the replication-competent avian sarcoma-leukosis virus to deliver oncogenes of interest to somatic cells engineered to express the t-va receptor. This system has been used successfully to engineer both pediatric brainstem glioma and adult glioma models (50–53). *In utero* electroporation is a distinct methodology that has been used successfully by a number of groups to create pediatric brain tumor models (10, 46, 54–58). This methodology has been found to be particularly useful in generating tumors that are characteristically located in highly eloquent regions such as the brainstem, i.e. diffuse intrinsic pontine glioma. In this technique, embryos from pregnant mice are identified following laparotomy, and plasmid DNA of interest is injected into the desired region of the brain. The region of the brain is then stimulated with pulsed electroporation to facilitate DNA uptake (54–58). Transposon-based mutagenesis has also been employed by multiple groups; the *Sleeping Beauty* transposon system is the most widely used in brain tumor modeling and a detailed description of this technique can be found elsewhere (47, 48, 59–61). Briefly, this system enables insertional mutagenesis of genes of interest into specific tissues to produce different types of tumors. One advantage of this approach is that this system can be used to perform functional genomic screens whereby genetic alterations suspected to be fundamental to tumorigenesis of various tumor types can be introduced and subsequently assessed through *in vitro* and *in vivo* assays (47, 48, 59–61).In one of the earliest GEMMs, *Reilly et al.* create an *Nf1*, *Trp53* compound heterozygote strain to create a range of astrocytomas driven by loss of *Trp53* tumor suppressor function (62). *Larson et al.* apply a similar approach to diffuse intrinsic pontine gliomas by creating a mouse model with neonatal H3.3K27M, *PDFRA* activation, and *Trp53* loss (63). They discover that together, these genetic alterations accelerate brainstem glioma formation signifying that these genes are implicated in the basic neurodevelopment of diffuse intrinsic pontine glioma (63). *Pathania et al.* created a similar model requiring only H3.3K27M and *Trp53* loss with *in utero* electroporation and mutagenesis (56). Groups have used these models for successful testing of epigenetic immunotherapeutic agents and small molecule inhibitors (43, 64). Similar models have also been developed for subtypes of medulloblastoma allowing development of mutation-specific therapies (28, 31, 65). Despite the utility of GEMMs in study key genetic alterations and tumorigenesis, certain models are limited by inability to recapitulate the true intratumoral and locoregional heterogeneity observed in human pediatric brain tumors (10, 41).

**Table 2:** Methodologies for generating brain tumor genetically engineered mouse models (GEMM).

Methodology	Representative study	Tumor type	Features	Therapeutics evaluated
*Cross-breeding*	(62) *Reilly et al.*	LGG, HGG	Recapitulated human glioma histology and grades	N/A
*Viral vector-based engineering*	(53) *Halvorson et al.* *(* 52) *Becher et al.*	DIPG	Recapitulated human DIPG histology and genetics	Small molecule multi-kinase inhibitor, radiation therapy
*In utero electroporation*	(57) *Patel et al.*	DIPG	Recapitulates human DIPG histology and molecular features	N/A
*Transposon-mediated delivery*	(59) *Beckmann et al.*	MB, PNET	Genetically induced model of PNET, group 3/group 4 MB *Sleeping Beauty* transposon system	N/A

LGG, low-grade glioma; HGG, high-grade glioma; DIPG, diffuse intrinsic pontine glioma; MB, medulloblastoma; PNET, primitive neuroectodermal tumor; NA, not applicable.

Murine xenograft models offer the closest opportunity to using human tumor and human immune system components for immunotherapy testing. Xenograft models are typically generated by isolated tumor cells from a patient and then transplanting them into an immunodeficient mouse line systemically or orthotopically (10, 41). Subsequently, *in vivo* studies may be performed with local or peripheral delivery of the immunotherapeutic agent or cell therapy. The major limitation with this type of model is the absence of a complete immune system which prevents study of how the immunotherapy being administered may interact with the tumor immune microenvironment. Groups have attempted to mitigate this limitation by reconstituting the mouse immune system with human stem cell and immune cell fractions (4, 66, 67). Development of several patient-derived xenograft banks are ongoing which will minimize the time investment and troubleshooting needed to isolate and grow patient cell lines (39, 65, 67–70).

Animal models are indispensable for studying immunotherapy efficacy in different contexts including interaction with the remainder of the tumor immune microenvironment (e.g. syngeneic model, humanized xenograft model) or epigenome-based immunotherapy surrounding fundamental genetic aberrations (GEMMs). However, they fail to provide granular information and insight into the cellular and molecular level interactions between immunotherapy and tumor immune microenvironment components such as the blood-brain barrier, extracellular matrix, and individual cell types (macrophages, myeloid-derived cells, etc.) (10, 67, 69). To interrogate these interactions, new models that mimic the brain architecture and physiological milieu are being investigated (10, 18, 20, 21, 37).

## 4 Physiomimetic preclinical modeling to recapitulate the tumor immune microenvironment

A significant obstacle to immunotherapy development is the lack of understanding of how different components of the tumor microenvironment and immune system interact with the proposed therapies. Recent advances in bioengineering and genetic engineering have paved the way for novel, hybrid models pediatric brain cancer that aim to deconstruct the heterogeneity and complexity of the tumor immune microenvironment and enable interrogation of individual interactions in a modular way (**Figure 3**) (10, 21, 36, 37, 65, 71, 72). Studies have demonstrated that there is considerable variability and heterogeneity between and within tumor types that extends to not only the immune milieu and cellular composition found with tumors, but also mechanical properties such as stiffness of the microenvironment related to the extracellular matrix and differential blood-brain barrier disruption (18, 21, 31, 38). For example, *Phoenix et al.* demonstrate the WNT-medulloblastoma compared to SHH-medulloblastoma does not form an intact blood-brain barrier due to aberrant paracrine signaling and this influences sensitivity and therapeutic response to cytotoxic chemotherapy (31). Similar findings have been observed in the extracellular matrix composition of tumors including high-grade gliomas and ependymomas that may promote angiogenesis and impede migration of anti-tumor factors and cell types to penetrate the tumor via a tenascin C-mediated mechanism (21, 73). In effort to understand the influence of these features that are not captured in convention, 2-D *in vitro* culture or cannot be modulated in a manner that is easy to study in animal models, groups have sought to develop systems that recapitulate the 3-D tumor microenvironment that includes cell populations and environmental factors (65, 70, 74).

Bioengineered 3-D systems represent one method to design a complex *in vivo* culture system that captures interactions between tumor cells and other cell types, both immune and non-immune, observed in tumors as well as the mechanical factors of the microenvironment. *Sood et al.* describe a 3-D extracellular matrix environment for the study of both pediatric ependymoma and glioblastoma (74). This system is comprised of a central hydrogel surrounded by a scaffold that can house other cell types including immune cells, astrocytes, and neurons. They implant primary, patient derived tumor cells into the central core and can implant other cell types as co-cultures to selectively interrogate interactions between cells or interactions between cells and the hydrogel matrix whose properties can also be altered (74). Such a platform also enables molecular and metabolic analysis of tumor cells and surrounding cells over different times points to capture temporal heterogeneity and better understand tumor evolution mechanisms in response to immunotherapies (74). *Tang et al.* show that a similar 3-D system can be bioprinted incorporating patient-derived glioma stem cells and cell types found in the tumor milieu including macrophages and astrocytes in a hydrogel comprised primarily of hyaluronic acid (75). They perform transcriptional profiling as well as assays evaluating immune activation and find that this model effectively recapitulates the immune and transcriptional phenotypes observed in human glioblastoma (75). Bioengineered 3-D systems offer a platform for sophisticated, high-throughput screening of therapeutics and with the incorporation of immune cells may be amenable to testing of immunotherapeutic agents and cell therapies in a similar fashion. Several groups have implemented the use of organotypic brain slice cultures to study functional characteristics of tumor cells including therapeutic sensitivities and migration/invasion potential (76–79). In this hybrid system, murine or patient-derived cell lines are implanted onto an *ex vivo* animal brain slice and observed over a predetermined time course to study these properties that are not easily quantified in animal models or other *in vitro* systems (76–79). This method carries the advantage of being a personalized assay if patient-derived tumor cells are used and has the ability to also assess efficacy and targeting of cell-based therapies (76, 79). Groups have employed organotypic models to study tropism of anti-tumor mesenchymal stem cell therapies for example and such an approach may be applied to immune-based cell therapies (77).

3-D and *ex vivo* model systems are effective for dissecting specific interactions and are relatively cost-efficient compared to other systems. However, these models typically cannot recapitulate intratumoral diversity or specific genetic alterations that are determined primarily by the cell lines used to populate the model (10, 21, 36, 37, 80). Organoid models of brain tumors represent a promising system that overcomes this limitation and also potentially preserves native cell-cell interactions (36, 37). *Jacob et al.* devise a methodology for the isolation of patient-derived glioblastoma organoids that avoids dissociation into single-cell culture to preserve native interactions and cytoarchitecture (80). Through their process, they perform extensive molecular and histopathological characterization of the organoid to demonstrate effective recapitulation of intra-tumoral and inter-tumoral heterogeneity. They also demonstrate that such a system is readily accessible for evaluation of immunotherapies and specifically CAR T cell therapies (80). By co-culturing EGFRvIII-specific CAR T cells with glioblastoma organoid, they demonstrate that clinically relevant parameters are able to observed including tumor cell death, persistence/expansion of the CAR T cells, and antigen escape phenomenon owing to the heterogeneity recapitulated by the organoid (80). *Lago et al.* achieve similar findings using patient-derived organoids of pediatric brain tumors including medulloblastoma and ependymoma using a methodology that involved dissociation of the collected tumor bulk into a single-cell suspension (81). Still, they showed that these organoids maintained their genetic and epigenetic integrity compared to patient samples and also displayed *bona fide* cellular heterogeneity (81). As a result, organoids may offer a robust platform for immunotherapy development and testing. Multiple studies have including those of brain cancer have reported the feasibility of organoid-immune co-cultures highlighting the capacity of this technology to study important interactions with the therapy being administered (21, 32, 36, 37). Due to the tunable nature of the system, organoids can be made increasingly complex to fulfill the needs of the preclinical testing which may involve complex co-cultures with immunotherapeutic agents, tumor cells, and multiple resident cell populations including macrophages and microglia (32, 36, 37, 80, 81). When patient-derived samples are used for these components, a high-throughput system can be designed to evaluate specific features of the immunotherapeutic agent or cell therapy of interest such as effectiveness of targeting (antibody-based therapies), persistence/exhaustion phenomenon (CAR T cell therapy), and neo-antigen enrichment or escape (epigenome-targeted therapies) (80). Moreover, the organoid culture technique can also be varied and can range from microfluidic-based systems to organ-on-a-chip systems that can be selected based on the needs of the testing (32, 36, 37). Organoids can be limited when incorporating tumor microenvironment features such as vascularity which may not be as robust as animal models or 3-D models, but efforts are underway to begin to develop these features as well (32, 36, 37).

## 5 Conclusion

Effective and multimodal preclinical modeling can help to optimize immunotherapy development before clinical translation. The sophistication of modeling has tremendously increased over the past years enabling interrogation of immunotherapy-tumor cell or immunotherapy-microenvironment interactions that were not easily achieved in the past. Leverage of newer technologies including organoids and advanced animal models in combination represents an important opportunity to streamline the immunotherapy development pipeline and detect potential areas of therapy failure or treatment resistance prior to clinical translation.

## 6 Discussion

**Figure 4:**
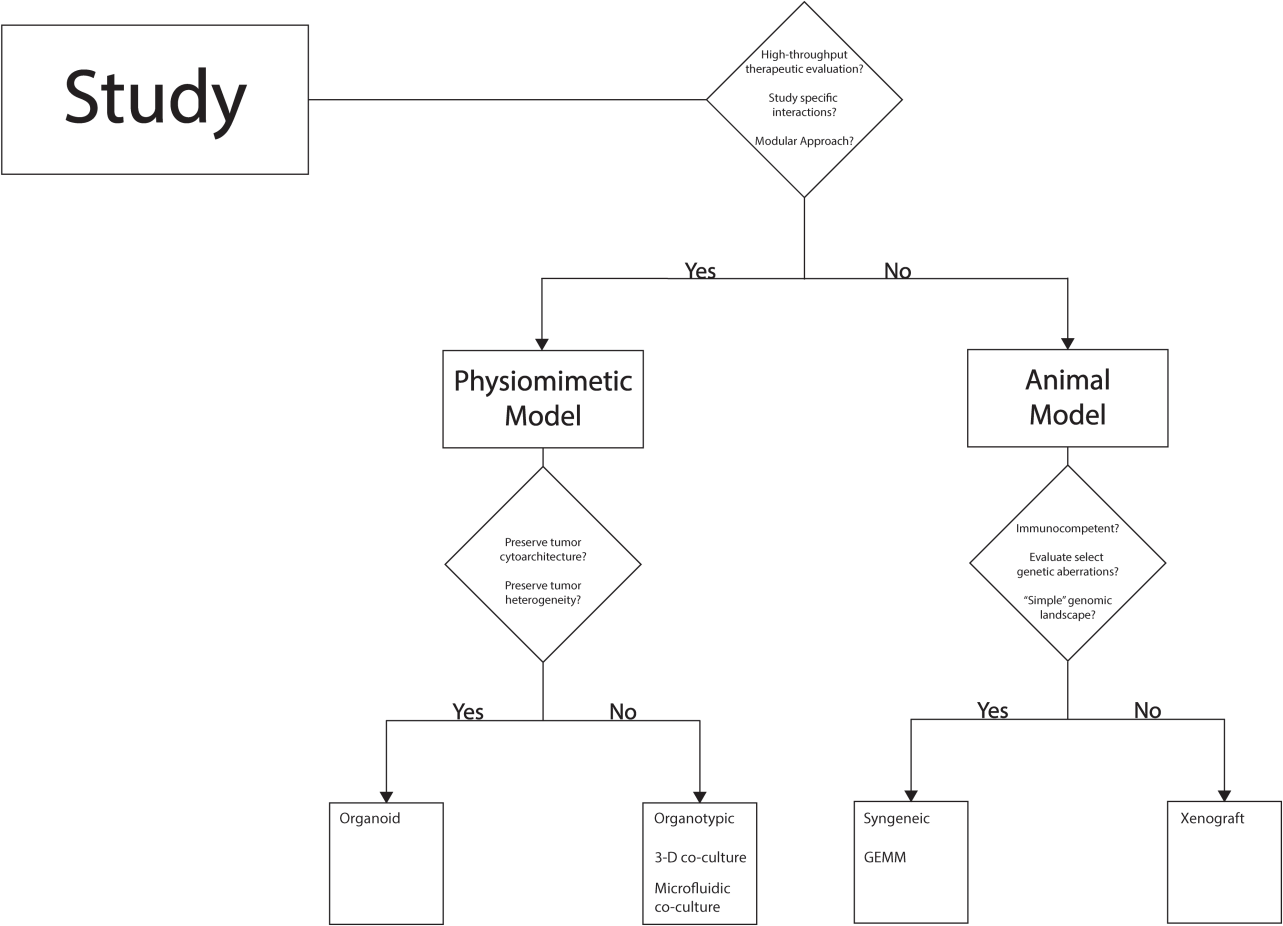
Decision-tree to guide selection of preclinical model.

Though significant advances in preclinical modeling of pediatric brain tumors have been realized in recent years, there continues to be gaps in the features of the tumor microenvironment and the immune microenvironment currently. As a result, there is no individual modeling strategy that is capable of accounting for the complexity of brain tumors pertaining to therapy development, so a thoughtful approach needs to be adopted in selecting a modeling strategy to effectively answer the question at hand while considering tumor-specific nuances. **Figure 4** offers one such framework to organize research questions and other considerations in selecting a modeling strategy. Certain strategies are particularly suited for exploratory studies within the context of pediatric neuro-oncology including high-throughput evaluation of therapeutic studies or studies looking to assess rudimentary efficacy based on a hypothesized therapeutic sensitivity or vulnerability. In such cases, *in vitro* studies utilizing readily available tumor cell lines is one option which is outside of the scope of the present review, but discussion of this strategy can be found elsewhere (10). A more sophisticated option in light of recent advances is the use of physiomimetic models including organoids as previously discussed. These models are much less time-consuming and labor intensive than animal models and are ripe for high-throughput study of therapeutics. Moreover, this category of models can be valuable in early mechanistic studies of tumor biology or therapeutic evaluation where a modular approach is used, and a single variable can be altered to interrogate a specific pathway or therapeutic sensitivity. For example, if the interaction of a tumor cell and a specific immune cell needs to be studied in the context of a therapy or a change in the mechanical microenvironment, an organoid model or organotypic model may be ideal. Conversely, if the research goal is to study the therapeutic efficacy of a well-characterized therapeutic strategy in anticipation of short-term clinical translation, animal models would be necessary to capture the complexity of the brain milieu and systemic immune system. Selection of the type of animal model can vary with the type of tumor being studied and whether the goal is to solely understand a feature of disease process or to also test a therapeutic agent, i.e. survival, adverse effects, etc. Certain tumor types such as DIPG that harbor clearly defined genetic or molecular events underlying tumorigenesis may be accurately modeled by strategies that can introduce these few aberrations efficiently (GEMM, syngeneic models) (4, 25, 43, 45, 57). Other tumor types such as glioblastoma or ependymoma tend to have a less “clean” genetic profile or series of critical events leading to tumorigenesis; in these cases, a xenograft model may be more feasible to capture the genomic and molecular complexity of the tumor and use alternative models when evaluating therapeutics (4, 23, 25, 80, 82–84). As technology and understanding of the basic biology of various pediatric brain tumors improve, a pipeline that incorporates multiple models may be feasible to leverage the strengths of each to investigate a mechanistic feature of the disease or a potential therapeutic in a streamline fashion.
